# The cumulative impact of health insurance on health status

**DOI:** 10.1111/1475-6773.13325

**Published:** 2020-07-22

**Authors:** Abigail R. Barker, Linda Li

**Affiliations:** ^1^ Brown School Washington University in St. Louis St. Louis Missouri USA

**Keywords:** health care costs, health insurance, health status, Medicaid, Medicare, race factors

## Abstract

**Objective:**

To add to the evidence base on causal linkages between health insurance coverage and health status, controlling for sociodemographic factors, by analyzing longitudinal data.

**Data Source:**

Secondary data from the Panel Study of Income Dynamics (PSID), 2009‐17, which is a longitudinal, multigenerational study covering a wide array of socioeconomic topics that began in 1968 but has only recently begun collecting useful information on individual health insurance.

**Study Design:**

2017 data on self‐reported health status, work limitations, and death were analyzed as outcomes based upon the degree of exposure to health insurance in 2011‐17. All variables were collected biannually for four years beginning in 2011. Having health insurance at each point in time was, in turn, modeled as a function of several sociodemographic factors.

**Data Extraction Methods:**

Data were downloaded using the crosswalk tool available at the PSID website. Because individual health insurance questions were only asked of heads and spouses in households beginning in 2011, we analyzed only these records.

**Principal Findings:**

Among respondents who were not in fair or poor health in 2009, each additional 2 years of subsequent reported insurance coverage reduced the chance of reporting fair or poor health in 2017 by 10 percent; however, this effect was not present for black respondents.

**Conclusions:**

Our results suggest that the effect of health insurance on health status may compound over time, although unevenly by race. Since people who report fair or poor health status represent the bulk of utilization and spending, our findings provide evidence in support of viewing coverage expansions as investments that will pay dividends in the form of lower utilization over time. More work is needed to produce detailed estimates of cost savings, which may in turn influence policy, as well as to understand and address the source of racial disparity.

**What is already known**

- A large and growing literature finds a positive association between health status and self‐reported health, using relatively short study periods, population subgroups, and survey data at the county level.

**What this study adds**

- Controlling for demographics including age, poverty, and education, we find evidence of a “dose/response” relationship between consistent health insurance coverage and self‐reported health status in our sample of all adults, although the relationship is not present for black respondents.
- Each additional two years of health coverage is associated with about a 10 percent reduction in the chance that an individual will fall into fair or poor health.
- This finding suggests that policies that support increased access to health insurance may help people maintain better health over time.

## 1 INTRODUCTION

While it is a compelling narrative that health insurance, by ensuring uninterrupted access to health care and in particular early access to preventive care, is likely to have a positive impact on health,^1^ much of the available evidence is unable to directly answer this question. Commentary by Levy and Meltzer in 2008,^2^ referring to their systematic review of the literature in 2004,^3^ stated that causality has not been firmly established; they suggested at that time that large investments in health and social programs would be needed to demonstrate causality. Subsequent studies using the longitudinal Health and Retirement Survey have examined the effects of health insurance coverage on health. In one study, continuously and intermittently uninsured adults ages 50‐60 were found to be more likely to have a major decline in overall health (defined by self‐reported health status) and the development of new physical difficulties compared with their continuously insured counterparts.^4^ Another study examined the effects of gaining Medicare at age 65 among people who were insured continuously prior to that time, compared with those who spent time uninsured before enrolling. Researchers found gains in health over multiple years among people who had been previously uninsured, compared with those who had always had coverage, especially for individuals with diabetes and cardiovascular disease.^5^ It was also calculated that public spending to insure individuals prior to age 65 would be offset by about half from savings due to improved health.^6^

In a systematic review of the causal effect of health insurance on outcomes in adults, Freeman et al conclude that health insurance improves health.^7^ However, the authors note that studies they identified in their review assessed the effect of insurance among individuals who were already sick. Similarly, in the 1971‐1982 large‐scale randomized RAND Health Insurance Experiment, the poorest and sickest sample at the start of the experiment were found to have better health outcomes under the free plan for four out of the thirty conditions measured compared to their counterparts with cost‐sharing. However, for the average person, no difference was found in health outcomes.^8^ Results from the more recent 2008 randomized control trial, the Oregon Health Insurance Experiment, support these findings in that no significant effect of gaining Medicaid coverage was found on clinical outcomes or mortality in the years following the lottery. Researchers did however find that the group assigned to health insurance coverage had better self‐reported physical and mental health compared with the control group, supporting results from longitudinal studies discussed above.^9^, ^10^ Recent results from a randomized pilot study in which the IRS sent informational letters to 3.9 million taxpayers who paid a tax penalty for lacking health insurance coverage under the Affordable Care Act found that the increase in coverage in the two years following reduced mortality among middle‐aged adults.^11^ Additionally, new evidence on Medicaid expansion supports the conclusion that new access to Medicaid among low‐income adults is significantly associated with reduced mortality, improved coverage, access to care, and self‐reported health. The authors discuss that their findings support a “plausible causal chain,” but do not use causal language, as the secondary outcomes of the study were not based upon individual‐level data. (The percentages of persons with Medicaid and in “excellent” or “very good” health were compared with the percentages without any health insurance.)^12^ Moreover, the study examines three Medicaid expansion states compared with three control states and focuses on the impact within the low‐income population, which may limit generalizability to the entire US population. The effect of health insurance coverage on health outcomes has been mixed, with most prior studies evaluating the relationship over a shorter time frame and for specific populations.

The objective of this work was to add to the evidence base on causal linkages between health insurance coverage and health status, controlling for sociodemographic factors, by analyzing individual‐level longitudinal data that are representative of the entire US population over a time horizon sufficient to detect a benefit if one exists. In particular, while it may be argued that there is a selection effect in that people choose to purchase—or not—health insurance coverage, the reality is that for most Americans, the choice is severely limited by one's education (making one eligible for the type of job that is likely to provide employer sponsored coverage) and one's income (as private market plans are not affordable for many higher‐income individuals, and a very low income qualifies a person for Medicaid coverage). Therefore, controlling for education and income, in particular, should be helpful in correcting for the selection effect. The longitudinal nature of the data helps us separate out the competing explanations for an association between health insurance and health: especially prior to the ACA, a person might become sick and then become uninsured either due to becoming unemployed or being denied coverage. The PSID helps identify such a chronology while also providing a cumulative health insurance variable that essentially allows us to capture a dose/response effect.

Evidence from the 2017 Medical Expenditure Panel Survey shows that self‐reported health status is highly correlated with total medical spending. Expenses incurred by those in excellent or very good health in 2017 averaged $3029; by those in good health averaged $6,545; and by those in fair or poor health averaged $16,670. A decline from “good” to “fair” doubled the average spending from $6545 to $13,918,^13^ so any policy that reduces the chance that an individual's health becomes “fair” or “poor” is likely to be associated with significantly less utilization and cost.

## 2 METHODS

The study was observational, using secondary data on self‐reported health status, work limitations, and death. These variables were analyzed as outcomes in a set of logistic models in which the degree of exposure to health insurance in 2011‐17 is the independent variable of interest. The models controlled for additional sociodemographic variables, including sex, race (white, black, and other race), educational status (high school less, some college, college degree or more), and poverty level. All variables were collected biannually for four years beginning in 2011. Having health insurance at each point in time was, in turn, modeled as a function of several sociodemographic factors. The data are from the Panel Study of Income Dynamics (PSID), 2009‐17, which is a longitudinal, multigenerational study covering a wide array of socioeconomic topics that began in 1968 but has only recently begun collecting useful information on individual health insurance. Data were downloaded using the crosswalk tool available at the PSID website.^14^ Because individual health insurance questions were only asked of heads and spouses in households beginning in 2011, we analyzed only these records, creating a total sample size of 9744. The specific question asked was “Do you currently have health insurance?” In each model, the sample was restricted to those with nonmissing data for the outcome variable and regressors.

Certain variable translations were made. Because health care utilization and expenditures increase significantly for individuals reporting “fair” and “poor” health,^15^ we combined these categories to create an outcome variable for being in either fair or poor health, vs. responding that one's health was “good,” “very good,” or “excellent.” For the insurance and poverty status variables, an adjustment was necessary due to some members of the sample dying during the sampling period. Therefore, we created a “percent of time insured” variable that equals 100 if the individual was insured over all 4 observation years—or if they were insured for all of the years in which they were alive. If they were insured for half of the years that they were alive, the variable equaled 50. If death occurred after 3 years, and the individual was insured for 1 of 3 years, then the variable equaled 33. A similar variable was constructed based upon the amount of time, in years, that an individual's annual income was above the federal poverty level (FPL). Someone alive all 4 years, with income below 100 percent FPL in 2 of the years, would have a value of 50. An individual who died after 3 years but lived in poverty for none of them would have a value of 100. These variables are constructed such that higher values correspond to better circumstances and are therefore hypothesized to reduce the chances of an individual falling into fair or poor health.

We used SAS PROC SURVEYLOGISTIC (SAS Enterprise Guide version 7.15) and applied appropriate sampling weights (which correct for oversampling and attrition) to estimate a set of logistic models to determine whether the degree of exposure to health insurance between 2011 and 2017 affected the chance that a person would rate their health in 2017 as fair or poor. We ran unconditional and conditional models; in the latter, we subset our sample to include only individuals who were not in fair or poor health in 2009. Similar models were run for the outcome variables of death (by 2017) and reporting of health limitations in ability to perform work in 2017.

## 3 RESULTS

Table 1 displays characteristics of the 9064 PSID respondents with nonmissing data in 2009 and who either had nonmissing data in 2011‐17 or who died in one of those years. Of these, 53 percent were females, while 82 percent were white, 13 percent were black, and 5 percent identified as another race. About 43 percent had a high school education or less, while 33 percent had a college degree or more education. In 2009, more than half (52 percent) reported being in excellent or very good health, while only 17 percent reported fair or poor health. A sizeable majority (79 percent) were insured for all four measurement years, with 19 percent experiencing partial coverage over time and only 2 percent being continuously uninsured in all years. Similarly, a large majority (81 percent) had incomes above the federal poverty level in all four years, while 16 percent had incomes that fluctuated above and below, and only 2 percent were consistently in poverty in all years. Table 2 further breaks down the sample by displaying the distribution of self‐reported health status categories for each demographic group. It shows that females, blacks, and those with less education and more consistent low incomes are somewhat more likely to have been in fair or poor health in 2009, which highlights the importance of comparing unconditional models with those conditioned on not being in fair or poor health in 2009.

**Table 1:** Characteristics of PSID sample population, weighted^a^

	% or Mean (SD) (*N* = 9744)	% or Mean (SD) nonmissing (*N* = 9065)
Age, 2009	51.0 (16.8)	50.3 (16.3)
Female	53.0%	53.0%
Race, %		
White	81.9	81.9
Black/African American	12.9	12.9
Other	5.2	5.3
Educational level, %		
High school or less	43.3	42.4
Some college	24.1	24.4
College or above	32.6	33.2
Percent of time income > 100%FPL, 2009‐2017		
0%	2.8	2.4
20%‐50%	5.1	5.1
51%‐80%	10.9	11.1
100%	81.2	81.4
Percent of time insured 2009‐2017		
0%	2.4	2.3
25%‐50%	9.7	9.7
51%‐75%	8.8	8.8
100%	79.0	79.2
Self‐reported health status in 2009, %		
Poor/Fair	18.1	16.6
Good	31.3	31.6
Very good/Excellent	50.6	51.8

^a^ Applied PSID sample weights to correct for oversampling and attrition over time to obtain estimates representative of all US households.

**Table 2:** Characteristics of PSID sample by self‐reported health status in 2009, weighted^a^

	Self‐reported health status in 2009 (*N* = 9,065)		
	Poor/Fair	Good	Very good/excellent
Mean (SD) Age in 2009	57.1 (16.4)	51.4 (15.9)	47.4 (15.8)
Sex, %			
Male	13.6	30.3	56.2
Female	19.2	32.8	48.0
Race, %			
White	15.3	30.7	54.0
Black/African American	24.2	35.3	40.5
Other	17.7	36.6	45.7
Education level, %			
High school or less	26.0	35.3	38.7
Some college	12.6	33.3	54.2
College or above	7.5	25.6	66.9
Percent of time income > 100%FPL, 2009‐2017			
0%	46.7	30.1	23.2
20%‐50%	39.1	35.4	25.5
51%‐80%	24.1	32.8	43.1
100%	13.2	31.3	55.5
Percent of time insured 2009‐2017			
0%	18.9	43.2	37.9
25%‐50%	16.7	35.6	47.7
51%‐75%	18.2	31.8	50.1
100%	16.3	30.8	53.0

^a^ Applied PSID sample weights to correct for oversampling and attrition over time to obtain estimates representative of all US households. All differences in health status are statistically significantly different (*P* < .0001) within each sociodemographic subcategory.

**Figure 1:**
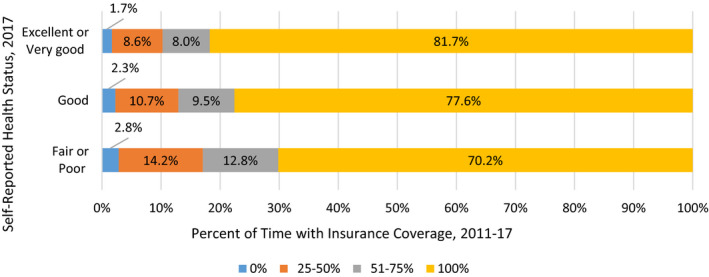
Self‐reported health status by degree of prior insurance coverage, 2017 (separate PDF) [Color figure can be viewed at wileyonlinelibrary.com]

The bivariate relationship between continuous insurance coverage over time and later health status is demonstrated in Figure 1. About 81.7 percent of those reporting excellent or very good health in 2017 had been insured in all four prior survey years, compared with 70.2 percent of those reporting fair or poor health in 2017. Furthermore, about 16.6 percent of those in excellent or very good health had been partially insured in the prior survey years, compared with 27.0 percent of those in fair or poor health. These findings are suggestive, but since insurance status is strongly correlated with employment and income and other social determinants, we turn to our multivariate findings.

First, the unconditional models consider each negative health outcome as predicted by a set of sociodemographic factors, plus the individual's status in terms of that outcome in 2009 (Table 3). Being in fair or poor health in 2009 was associated with an eightfold increase in the chance of fair or poor health in 2017, with more than a threefold increase in the chance of death. Reporting a work limitation in 2009 increased the chance of a work limitation in 2017 almost 12‐fold. Regardless of the specific negative health outcome considered, age was always significantly associated with negative outcomes. Each additional year of age increased the chance of fair or poor health in 2017 by 2.4 percent, of death by 2017 by 8.8 percent, and of a limitation in the ability to work in 2017 by 4.0 percent. Females in the sample in 2009 were only 61.2 percent as likely to die by 2017 as males were, and they were only 90.5 percent as likely to report a work limitation in 2017. Racial identity was significant only in predicting fair or poor health status, with blacks being 18.9 percent more likely than whites to report fair or poor health in 2017, and respondents of other races being 44.3 percent more likely to report fair or poor health in 2017. Education had a protective effect, where those who held college degrees or above were only 48.4 percent as likely to report fair or poor health in 2017 as those with no more than a high school education. Similarly, the most educated group was 63.5 percent as likely to die by 2017 and only 55.6 percent as likely to report a work limitation in 2017 compared with the least educated group. Poverty status was also significant, with each additional percentage point of the time between 2011 and 2017 that the individual's household income was greater than the poverty level being associated with about a 0.9 percent reduction in the chance of fair or poor health in 2017. The estimates were similar for the other outcome variables. Finally, each additional percentage point of time between 2011 and 2017 in which the individual was insured was associated with a 0.3 percent reduction in the chance of reporting fair or poor health in 2017. Evidence for the effect of health insurance coverage was mixed for the other two outcome variables, with no significant effect found on death by 2017 nor on the chance of reporting a work limitation.

**Table 3:** Unconditional odds of all outcomes in or by 2017, based upon 2009‐17 sociodemographics

	Fair or poor health in 2017	Death by 2017	Work limitation in 2017
	OR (95% CI)	OR (95% CI)	OR (95% CI)
Age	1.024 (1.019, 1.029)^a^	1.088 (1.077, 1.099)^a^	1.040 (1.034, 1.046)^a^
Gender (ref = Male)			
Female	1.058 (0.938, 1.194)	0.612 (0.473, 0.793)^a^	0.905 (0.761, 1.076)
Race (ref = White)			
Black/African American	1.189 (1.022, 1.384)^a^	0.948 (0.770, 1.167)	0.949 (0.792, 1.137)
Other	1.443 (1.021, 2.041)^a^	0.718 (0.401, 1.288)	0.797 (0.575, 1.105)
Educational level (ref = High school or below)			
Some college	0.808 (0.673, 0.971)^a^	0.797 (0.613, 1.038)	0.931 (0.813, 1.066)
College and above	0.484 (0.407, 0.576)^a^	0.635 (0.480, 0.841)^a^	0.556 (0.458, 0.675)^a^
Percent of time insured 2009‐2017	0.997 (0.995, 0.999)^a^	0.997 (0.990, 1.003)	1.003 (0.999, 1.006)
Percent of time income > 100% FPL, 2009‐2017	0.991 (0.989, 0.994)^a^	0.992 (0.988, 0.995)^a^	0.988 (0.985, 0.991)^a^
Fair or poor health in 2009	8.146 (7.008, 9.469)^a^	3.183 (2.469, 4.103)^a^	N/A
Work limitation in 2009	N/A	N/A	11.611 (10.182, 13.241)^a^

Note

The overall rate of fair or poor health increased from 18.1 percent in 2009 to 20.4 percent in 2017. The overall rate of having a work limitation increased from 19.9 percent in 2009 to 21.1 percent in 2017. All individuals in our sample were alive in 2009; the overall rate of death by 2017 was 11.4 percent. PSID sample weights to correct for oversampling and attrition over time were applied to obtain estimates representative of all US households.

^a^ Indicates significance at the alpha = 0.05 level. N/A means an explanatory variable is not included in a model.

Because the prior status of the outcome variables is such a strong predictor in the original models, we estimated a conditional model, in which we subset the data to those individuals who were not in fair or poor health in 2009. Conditional on health status being good, very good, or excellent in 2009, many variables continue to have similar effects on the chance of having fair or poor health in 2017 (Table 4, left column). The protective effect of education increases, as does the effect of income above the federal poverty level. In this conditional model, each additional percentage point of time covered by insurance is associated with a 0.4 percent reduction in the chance of fair or poor health in 2017. Stated in years of coverage, with one biannual observation corresponding to 25 percent of the study timeframe for individuals with complete data, this may be interpreted as an additional two years of coverage between 2011 and 2017 being associated with a 10 percent reduction in the chance of having fair or poor health in 2017.

**Table 4:** Conditional models of fair/poor health in 2017, given not in fair/poor health in 2009

	Fair or poor health in 2017			
	OR (95% CI), All	OR (95% CI), white only	OR (95% CI), black only	OR (95% CI), other only
Age	1.024 (1.018, 1.030)^a^	1.027 (1.020, 1.035)^a^	1.018 (1.008, 1.028)^a^	1.028 (1.013, 1.044)^a^
Gender (ref = Male)				
Female	1.013 (0.888, 1.156)	1.022 (0.847, 1.234)	0.977 (0.801, 1.193)	1.087 (0.737, 1.604)
Race (ref = White)				
Black/African American	1.198 (1.009, 1.423)^a^	N/A	N/A	N/A
Other	1.587 (1.049, 2.402)^a^	N/A	N/A	N/A
Educational level (ref = High school or below)				
Some college	0.792 (0.644, 0.973)^a^	0.820 (0.607, 1.107)	0.847 (0.677, 1.059)	0.377 (0.146, 0.971)^a^
College and above	0.435 (0.351, 0.540)^a^	0.419 (0.323, 0.543)^a^	0.618 (0.433, 0.884)^a^	0.398 (0.144, 1.100)
Percent of time insured 2009‐2017	0.996 (0.994, 0.999)^a^	0.992 (0.988, 0.996)^a^	1.001 (0.997, 1.005)	0.997 (0.988, 1.006)
Percent of time income > 100% FPL, 2009‐2017	0.990 (0.987, 0.992)^a^	0.979 (0.975, 0.984)^a^	0.995 (0.991, 0.998)^a^	0.989 (0.977, 1.000)^a^

Note

The overall rate of fair or poor health increased from 18.1 percent in 2009 to 20.4 percent in 2017. The overall rate of having a work limitation increased from 19.9 percent in 2009 to 21.1 percent in 2017. All individuals in our sample were alive in 2009; the overall rate of death by 2017 was 11.4 percent. PSID sample weights to correct for oversampling and attrition over time were applied to obtain estimates representative of all US households.

^a^ Indicates significance at the alpha = 0.05 level. N/A means an explanatory variable is not included in a model.

Because there was evidence of an interaction effect between racial identity and the amount of time covered by insurance, we ran separate analyses for whites only (Table 4, second column), blacks only (Table 4, third column), and other race only (Table 4, right column). These separate analyses show that the beneficial effects of health insurance suggested by the analysis of the full sample are entirely driven by the experience of whites in the sample. Each additional percentage point of the time with health insurance coverage was associated with a 0.8 percent reduction in the chance of whites reporting fair or poor health in 2017, but there was no significant effect (indeed, an insignificant gain) for black respondents. There was also no significant effect for other races.

## 4 DISCUSSION

This analysis of the PSID, a panel dataset with newly available individual‐level data on health insurance coverage in 2011‐17, provides evidence consistent with much previous work on the association between health insurance coverage and health status. Furthermore, due to the longitudinal nature of the data, and by controlling for sociodemographic covariates, we are able to provide new evidence that more consistent health insurance coverage over time decreases the probability that individuals will report fair or poor health at a later time. While there is still the potential for confounding due to selection effects (e.g. education is associated with better health and with higher income, thus making private health insurance more affordable), we have controlled for the most likely confounders in our models. Sub‐analyses on the population in good or better health in 2009 reinforced our finding. Similar findings for mortality as an outcome also suggested that a greater degree of insurance coverage over time lowered the risk of death by 2017. Since adverse selection, the traditional concern among economists that sicker individuals are more likely to purchase insurance, would tend to dilute any positive finding, we believe that our findings may be underestimates of the true impact of consistent health insurance coverage on health status and mortality.

Although we also examined the outcome of work limitations, we did not find a significant health insurance effect. This may be due to the co‐occurrence (within the same year) of work limitations and the availability of public insurance coverage through SSI Medicaid, SSDI Medicare, or both, although there is often a lag in obtaining Medicaid while waiting 2 years for Medicare. Depending on state policy, some applicants with work limitations might not qualify at all. Our measures were not granular enough to determine specific insurance type, nor which may have come first chronologically. However, we did run models comparing the early‐only insured (having insurance for the first 1‐2 periods only) vs. the late‐only insured (having insurance for the last 1‐2 periods only) and found that the early‐only group was about half as likely to report a work limitation in 2017. This strongly suggests that confounding due to health insurance eligibility due to work limitations is occurring.

Our analysis also revealed that the protective effect of health insurance on health status and mortality was mainly driven by the experiences of whites, and this is consistent with other recent research on the effect of Medicaid Expansion on health disparities. Expansion was found to be more effective in improving access and health outcomes among white low‐income childless adults and had relatively few favorable impacts on access and health outcomes for blacks and Hispanics.^16^ Social inequalities experienced as one is growing up have been found to relate to health and mortality across the adult life course.^17^ Our analysis does not include the effects of health insurance before 2011, yet health during childhood and adolescence are important precursors of health in adulthood.^18^, ^19^ Studies have found that health insurance during childhood, specifically Medicaid, positively affect health and predict future adult health outcomes.^20^, ^21^

Additional limitations of this research include the biannual measurement of all variables, given that health insurance status, as well as income, can fluctuate on a monthly or seasonal basis. It is unlikely that we have captured all of the interruptions of coverage that occurred, meaning that our estimate of the impact of truly continuous coverage on health is biased downward. In contrast, if those with unmeasured coverage interruptions are disproportionately likely to be low‐income or members of a minority race, then the model will mistakenly attribute some of the increased risk from this (unobserved) interruption to the person's demographic characteristics. Also, the PSID contains very complex and dynamic information about marital status that is not easily recoded into year‐to‐year indicators, so marital status was omitted as a possible explanatory variable. However, one purpose in using the PSID is that it is a population‐level dataset not focused only on Medicare‐ or Medicaid‐eligible populations, so its findings are more general than those of many other studies. A final limitation is that, although the approach for this analysis is intended to facilitate a causal interpretation, there may be additional sources of selection bias for which our data do not allow us to control. Like other authors, we therefore describe our work as providing additional evidence of a “plausible causal chain.”

Broadly, this paper finds that consistent health insurance is a driver of health. It suggests that other modifiable factors, such as education and income, are important drivers as well. These results support multi‐faceted social policy approaches to improving health. However, the most straightforward goal is likely that of increased access to health insurance coverage, whether through modifications to the Affordable Care Act, additional states taking up the Medicaid expansion option, or proposals that aim for universal coverage. There is a clear relationship between self‐reported health status and health care costs, and our findings provide evidence in support of viewing coverage expansions as investments that will pay dividends in the form of lower utilization over time. However, as such coverage gains may have uneven effects by race, additional policy options should also be considered to ensure that coverage gains translate to equal access to health care for all racial groups.

## CONFLICT OF INTEREST

No other disclosures.

## Supporting information

Author Matrix
